# Dynamic *in vivo* mutations within the *ica* operon during persistence of *Staphylococcus aureus* in the airways of cystic fibrosis patients

**DOI:** 10.1371/journal.ppat.1006024

**Published:** 2016-11-30

**Authors:** Bianca Schwartbeck, Johannes Birtel, Janina Treffon, Lars Langhanki, Alexander Mellmann, Devika Kale, Janina Kahl, Nina Hirschhausen, Claudia Neumann, Jean C. Lee, Friedrich Götz, Holger Rohde, Hanae Henke, Peter Küster, Georg Peters, Barbara C. Kahl

**Affiliations:** 1 Institute of Medical Microbiology, University Clinics Münster, Germany; 2 Channing Laboratory, Brigham and Women’s Hospital, Harvard Medical School Boston, United States of America; 3 Institute for Hygiene, University Clinics Münster, Germany; 4 Department of Microbial Genetics, University of Tübingen, Tübingen, Germany; 5 Institut für Medizinische Mikrobiologie, Virologie und Hygiene, Universitätsklinikum Hamburg-Eppendorf, Hamburg, Germany; 6 Pediatric Department Clemenshospital Münster, Münster, Germany; Columbia University, UNITED STATES

## Abstract

Cystic fibrosis (CF) is associated with chronic bacterial airway infections leading to lung insufficiency and decreased life expectancy. *Staphylococcus aureus* is one of the most prevalent pathogens isolated from the airways of CF patients. Mucoid colony morphology has been described for *Pseudomonas aeruginosa*, the most common pathogen in CF, but not for *S*. *aureus*. From the airways of 8 of 313 CF patients (2.5%) mucoid *S*. *aureus* isolates (n = 115) were cultured with a mean persistence of 29 months (range 1 month, 126 months). In contrast to non-mucoid *S*. *aureus*, mucoid isolates were strong biofilm formers. The upstream region of the *ica* operon, which encodes the proteins responsible for the synthesis of the polysaccharide intercellular adhesin (PIA), of mucoid isolates was sequenced. *Spa*-types of mucoid and non-mucoid strains were identical, but differed between patients. Mucoid isolates carried a 5 bp deletion in the intergenic region between *icaR* and *icaA*. During long-term persistence, from two patients subsequent non-mucoid isolates (n = 12) with 5 bp deletions were cultured, which did not produce biofilm. Sequencing of the entire *ica* operon identified compensatory mutations in various *ica*-genes including *icaA* (n = 7), *icaD* (n = 3) and *icaC* (n = 2). Six sequential isolates of each of these two patients with non-mucoid and mucoid phenotypes were subjected to whole genome sequencing revealing a very close relationship of the individual patient’s isolates. Transformation of strains with vectors expressing the respective wild-type genes restored mucoidy. In contrast to the non-mucoid phenotype, mucoid strains were protected against neutrophilic killing and survived better under starvation conditions. In conclusion, the special conditions present in CF airways seem to facilitate ongoing mutations in the *ica* operon during *S*. *aureus* persistence.

## Author Summary

*Staphylococcus aureus* is one of the most common pathogens isolated from the airways of cystic fibrosis (CF) patients. In this study, we identified unusual mucoid *S*. *aureus* isolates in 8 of 313 (2.5%) CF patients. All mucoid isolates carried a 5 bp deletion upstream of the *ica* operon, which resulted in increased expression of PIA/PNAG biofilm. In three patients, mucoid isolates were recovered for extended periods up to 126 months. Surprisingly, later sequential non-mucoid isolates (n = 12) of two patients also carried the 5 bp deletion. Sequencing of the entire *ica* operon identified compensatory mutations in different *ica* genes (*icaA*, *icaD*, *icaC*) in these isolates. A close relationship of these isolates and of the first mucoid and closest non-mucoid isolate without 5 bp deletion were confirmed by whole genome sequencing. Transformation with expression vectors with respective wild-type genes restored mucoidy. Mucoid isolates were protected against neutrophil killing and survived better under starvation conditions. In conclusion, the special conditions present in CF airways seem to facilitate ongoing mutations in the *ica* operon during persistence of *S*. *aureus*.

## Introduction

Cystic fibrosis (CF) is one of the most common hereditary diseases in the Caucasian population caused by mutations of an important chloride channel (cystic fibrosis transmembrane regulator) and affects worldwide approximately 70,000 people [1]. The mutation leads to impaired mucociliary clearance by airway epithelial cells with ensuing recurrent suppurative bacterial infections [2]. *Staphylococcus aureus* is one of the first and today the most frequent isolated pathogen, which can be recovered from the airways of CF patients with increasing prevalence rates most likely due to early eradication strategies directed against *Pseudomonas aeruginosa*, which was the leading pathogen in CF for decades and which has been shown to be responsible for lung function decline [1,3]. Mucoid isolates of P. *aeruginosa* occur in late stages of CF after the patients experienced long-term persistence of non-mucoid *P*. *aeruginosa* phenotypes [4]. The recovery of mucoid isolates has been shown to play a greater role in lung disease progression than the recovery of non-mucoid *P*. *aeruginosa* isolates [4]. The underlying mechanism for mucoidy is caused by overproduction of alginate due to a mutation in the *mucA* gene [5,6].

It has been shown that biofilm formation of *S*. *aureus* occurs in CF patients in vivo, which in part explains persistence of *S*. *aureus* in this particular niche [7,8]. Significantly, biofilm formation protects *S*. *aureus* from the host’s immune response and renders the species intrinsically more resistant against antibiotics [9]. Different mechanisms contribute to *S*. *aureus* cell aggregation and subsequent biofilm formation [10,11]. Of significant importance is the polysaccharide intercellular adhesin (PIA), also known as poly-N-acetyl-β-(1–6)-glucosamine (PNAG), which is encoded by the *icaADBC* locus and regulated by *icaR* located upstream of the *icaA* start codon [12]. The functions for the single *ica* genes are only partly resolved. There is biochemical evidence that IcaA functions as a N-acetylglucosaminyl transferase, and IcaD might be a chaperone that directs the correct folding and membrane insertion of IcaA [13]. No published evidence for the function of IcaC is available, but it has been speculated that the protein is involved in the externalization of PIA/PNAG [14], while IcaB acts as a deacetylase responsible for de-acetylation of mature PIA [15].

The *ica* operon is present in almost all isolates, but *S*. *aureus* usually only produces a scant biofilm under in vitro conditions [12]. Interestingly, *agr*-negative *S*. *aureus* strains produce higher amounts of biofilm [16]. Furthermore, Cramton et al. showed that anaerobic conditions as typically observed in mucus plugs within the airways of CF patients [17], induced the expression of PIA/PNAG [18]. Later, Ulrich et al. identified *SrrAB* as a major activator of *ica* expression under anaerobic conditions, resulting in protection of *S*. *aureus* against neutrophil killing under anaerobic conditions [19].

Recently, Jefferson et al. described a hyper-biofilm forming *S*. *aureus* strain, which was isolated accidently in the laboratory [20]. The authors identified a 5 bp deletion upstream of the *ica* operon and downstream of the *ica* repressor, which was responsible for this unusual phenotype [20].

Mucoid *S*. *aureus* phenotypes have not been described to occur in vivo or during chronic CF airway infection. Since we occasionally isolated unusual mucoid *S*. *aureus* isolates from some patients of the two CF centers in Münster, Germany, we aimed to determine the prevalence, persistence and underlying mechanism responsible for mucoidy.

Therefore, we retrospectively analyzed microbiological results of *S*. *aureus* strains collected from two independent prospective studies for the occurrence of mucoid isolates. Mucoid and non-mucoid isolates were characterized by *spa*- and MLST-typing, and biofilm formation was assessed using a static biofilm assay. The *icaR-icaA* intergenic region was sequenced to detect a possible 5 bp deletion [20], which could be responsible for the hyper-biofilm formation. Furthermore, the mucoid isolates were compared to non-mucoid isolates by competition experiments to assess for fitness loss, survival under starvation conditions to determine survival advantages and in opsonophagocytic assays to analyze protection against neutrophilic killing.

## Results

### Prevalence and persistence of mucoid *S*. *aureus* isolates from the airways of CF patients

**Fig 1:**
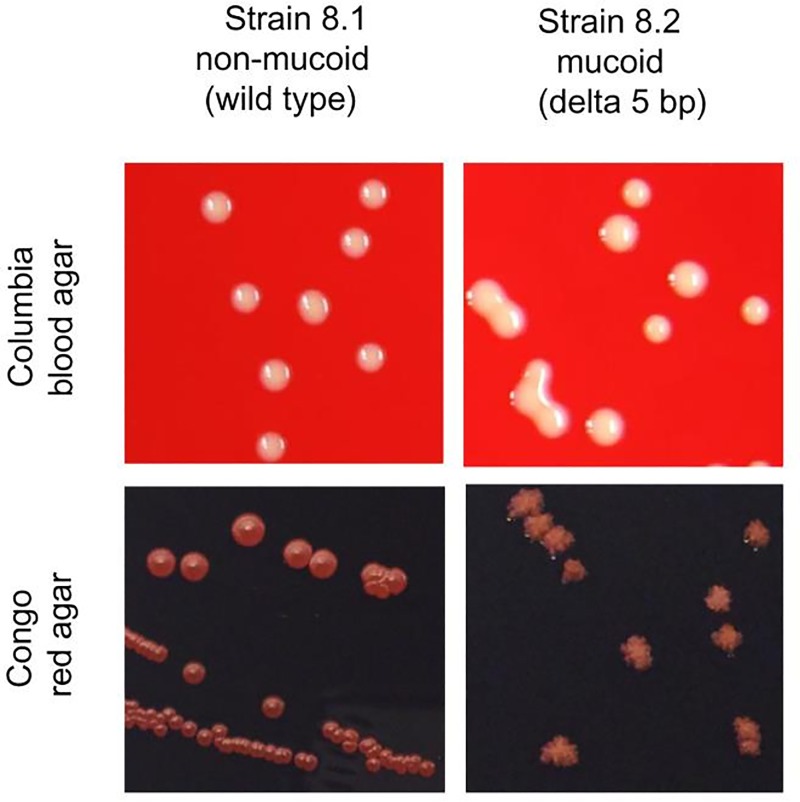
Morphology of non-mucoid and mucoid *S*. *aureus* on Columbia blood agar and Congo red agar (CRA). *S*. *aureus* strains were diluted in PBS and streaked on Columbia blood agar to observe single colonies. The macroscopic morphology of the non-mucoid and mucoid *S*. *aureus* isolates revealed significant differences in colony phenotypes. Whereas it was possible to isolate single colonies for the non-mucoid phenotype, the mucoid colonies stuck together, and it was almost impossible to recover bacteria from the agar. (See the movie in the supplemental data). On CRA, non-mucoid *S*. *aureus* displays smooth colonies, whereas mucoid isolates exhibit dry wrinkled colonies.

In our laboratory, airway cultures from CF patients occasionally yielded unusual mucoid *S*. *aureus* isolates, which are not necessarily detectable by eye on Columbia blood agar plates (Fig 1), but which are especially recognizable by the unusual sticky phenotype, which can be assessed by sub-culturing (Suppl. Material, movie). The mucoid phenotype is easily recognizable on Congo red agar (CRA) plates, because of the wrinkled dry colonies in contrast to smooth round colonies of the non-mucoid phenotype (Fig 1).

To determine the prevalence of mucoid *S*. *aureus* isolates in *S*. *aureus*-positive CF patients from two CF centers in Münster, Germany (n = 118), and from a recent prospective longitudinal multicenter study (n = 195), we reanalyzed our microbiological data and identified 115 mucoid isolates, which were isolated from nasal or throat swabs or sputa from 8 patients (7 male, median age at first mucoid isolate 24 years) with a mean persistence of 29 months (range: 1 month to 126 months, Table 1).

**Table 1:** Prevalence and persistence of mucoid *S*. *aureus* isolates in respiratory specimens of CF patients.

patients	gender	age at first mucoid isolate(years)	persistence of mucoid isolates(months)	number of allisolates^1^	number of mucoid isolates(% of all normal isolates)^2^	number of non-mucoid isolates with 5bp-deletion(% of mucoid isolates)^3^	*spa-*type^4^	MLST^5^	persistent *P*. *aeruginosa*
**1**	m	5	24	18	11 (61)	no	t087	ST25	no
**2**	m	34	1	12	4 (33)	no	t189	ST188	yes
**3**	m	34	3	34	4 (12)	no	t306	ST5	no
**4**	m	14	5	17	2 (12)	no	t012	ST30	no
**5**	f	21	1	20	2 (1)	no	t091	ST7	no
**6**	m	23	1	14	1 (1)	no	t2845	ST1909	no
**7**	m	24	68	76	27 (35)	6 (22)	t002	ST5	yes
**8**	m	27	126	234	64 (27)	8 (12)	t618	ST30	yes

^1^all isolates, which were cultured during the observation period

^2^all mucoid isolates during this period, in parenthesis the percentage of mucoid isolates compared to non-mucoid isolates

^3^in parenthesis, the percentage of the number of non-mucoid isolates with 5 bp deletion in comparison to the number of mucoid isolates with 5 bp deletion

^4^all investigated non-mucoid and mucoid *S*. *aureus* isolates revealed the same *spa*-type

^5^MLST analyses was only performed for one non-mucoid and mucoid *S*. *aureus* isolate of each patient. The same MLST was determined for the *S*. *aureus* isolates of every patient.

To determine clonality, we performed *spa*- and MLST-typing of all isolates, which indicated that non-mucoid and mucoid isolates belonged to the same *spa*-type in each patient, but differed between patients (Table 1) with two strains each belonging to ST5 and ST30. These data show that mucoid phenotypes are not restricted to a special *S*. *aureus* clone but can occur in different genetic *S*. *aureus* lineages.

### Capsule expression is not enhanced in the mucoid compared to the non-mucoid phenotype

*S*. *aureus* produces capsule polysaccharides (CP) also termed micro-capsules, which can be differentiated by rabbit serum antibodies [21]. These (CPs) represent an important virulence factor of *S*. *aureus* due to protection against phagocytosis. Most clinical *S*. *aureus* isolates belong to CP-type 5 or 8 [21]. Therefore, we hypothesized that hyper-expression of CPs could be responsible for the mucoid phenotype. We determined CP expression by a semi-quantitative colony immunoblot assay [22] for selected isolates of patient 8. Mucoid strains varied in their CP expression between weak and strong and did not differ significantly compared to the non-mucoid strains (S1 Table). Quantification of capsule production by ELISA inhibition confirmed the results of the colony immunoblot assays (S1 Fig), indicating that CP expression did not explain the mucoid phenotype of the isolates.

### Mucoid isolates are hyper-producers of PIA-associated biofilms

**Fig 2:**
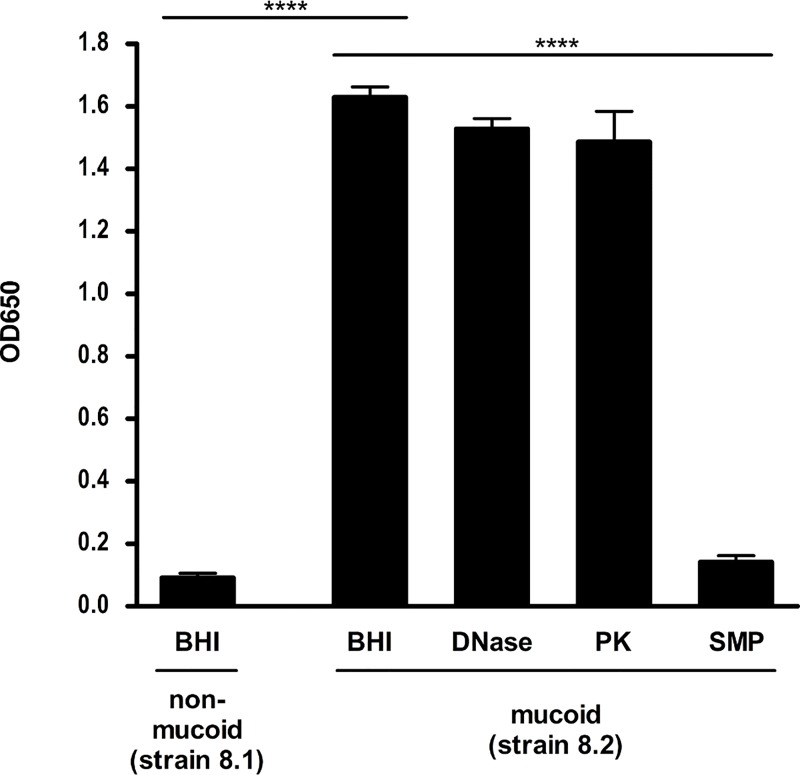
Biofilm formation of non-mucoid and mucoid phenotypes. A static biofilm assay was performed after overnight culture of the non-mucoid strain 8.1 and the isogenic mucoid *S*. *aureus* strain 8.2 (Table 2) of patient 8 in BHI containing 0.25% glucose, incubation for 24 h at 37°C and staining with 1% crystal violet. Biofilms were solubilized in ethanol-acetone and the absorbance was determined at OD_655nm_ with a microtiter plate reader. Each experiment was performed at least three times, and in each experiment the samples were prepared in eight replicate wells. To determine the nature of biofilm, detachment of the biofilm was measured after treatment of the wells, which have been cultured as described above, with sodium-metaperiodate, DNase or proteinase K before measuring the absorbance. All experiments were repeated three times. Results are shown as means and standard deviation. **** p-value ≤ 0.0001.

Another possible explanation for the mucoid phenotype could be overproduction of PIA/PNAG leading to enhanced biofilm formation. Assessment of biofilm formation using a conventional microtiter plate assay showed that all mucoid isolates displayed enhanced biofilm production, whereas almost no biofilm was formed by the non-mucoid strains as exemplified with strains 8.1 and 8.2 (Fig 2, Table 2). To investigate the nature of the biofilm, we treated the biofilm with sodium metaperiodate, which breaks down PIA/PNAG-dependent biofilms, and by proteinase K or DNase, which disrupts protein-or DNA-dependent biofilms, respectively. The biofilm of the mucoid isolate was dissolved by sodium metaperiodate, but not by proteinase K or DNase, indicating that PIA/PNAG is functionally involved in the biofilm formation (Fig 2).

**Table 2:** *S*. *aureus* isolates used in the studies with or without a 5 bp deletion exhibiting the non-mucoid or mucoid phenotype.

patient	isolate	date	*spa* type	phenotype	5 bp deletion	gene	location, related to#ATCC 35556	type of mutation in *ica*	transformation with plasmid	phenotype after transformation
7	7.1	18.01.2010	t002	non-mucoid	no					
	7.2	18.03.2010	t002	mucoid	yes					
	7.3	20.04.2010	t002	non-mucoid	no					
	7.4	06.01.2011	t002	non-mucoid	yes	*icaA*	2940–2941	2 bp insertion (GT)	pTXicaAD	mucoid
	7.5	06.01.2011	t002	non-mucoid	yes	*icaA*	2929–2940	12 bp deletion (TATTTCGGGTGT)	pTXicaAD pTXicaBC	mucoid non-mucoid
	7.6	14.03.2012	t002	non-mucoid	yes	*icaD*	3524	1 bp substitution (G → T)	pTXicaAD	mucoid
									pTXicaBC	non-mucoid
	7.7	11.06.2012	t002	non-mucoid	yes	*icaD*	3524	1 bp substitution (G → T)	pTXicaAD	mucoid
	7.8	19.06.2012	t002	non-mucoid	yes	*icaD*	3524	1 bp substitution (G → T)	pTXicaAD	mucoid
	7.9	09.10.2012	t002	non-mucoid	yes	*icaC*	5539–5542	4 bp deletion (TTTA)	pTXicaBC	mucoid
8	8.1	17.03.2005	t618	non-mucoid	no					
	8.2	17.03.2005	t618	mucoid	yes					
	8.3	12.09.2006	t618	non-mucoid	yes	*icaC*	4900	1 bp substitution (C → A)	pTXicaBC	mucoid
	8.4	02.04.2007	t618	non-mucoid	yes	*icaA*	3164	1 bp deletion (T)	pTXicaAD	mucoid
									pTXicaBC	non-mucoid
	8.5	02.04.2007	t618	non-mucoid	no					
	8.6	02.04.2007	t618	mucoid	yes					
	8.7	23.05.2008	t618	non-mucoid	yes	*icaA*	2954	1 bp deletion (A)	pTXicaAD	mucoid
									pTXicaBC	non-mucoid
	8.8	10.03.2014	t618	non-mucoid	yes	*icaA*	2954	1 bp deletion (A)	not done	
	8.9	15.10.2014	t618	non-mucoid	yes	*icaA*	2954	1 bp deletion (A)	not done	
	8.10	24.11.2014	t618	non-mucoid	yes	*icaA*	2954	1 bp deletion (A)	not done	

### Enhanced biofilm structures in mucoid isolates due to PIA/PNAG

**Fig 3:**
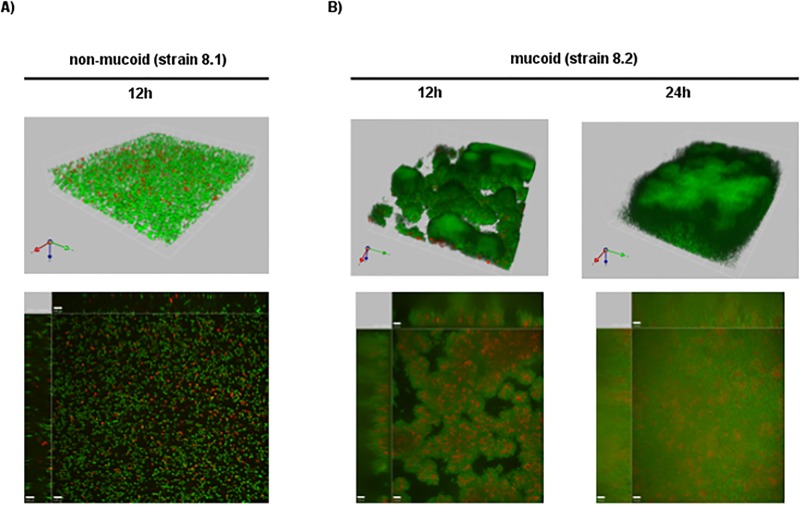
Confocal microscopy of biofilms of the non-mucoid (strain 8.1) and mucoid *S*. *aureus* (strain 8.2) phenotypes. Adherent bacteria were stained using the LIVE/DEAD *Bac*Light kit. A). The green color indicates live and the red color dead cells. Only scant amounts of biofilm were detected for non-mucoid isolates. The average biofilm thickness was low (<10 μM at 24h). B) The mucoid strain produced a stable biofilm, which revealed the typical mushroom-shaped multicellular structure. The average height of the biofilm produced by the mucoid strain was 23 μM after 24 h.

**Fig 4:**
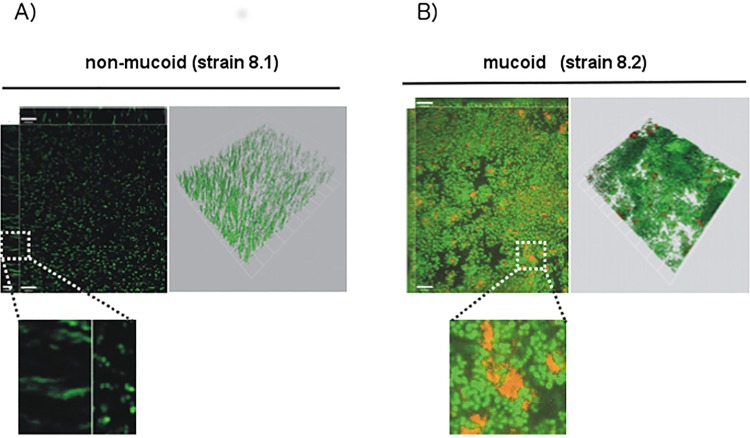
Confocal microscopy of biofilms stained with PIA/PNAG-specific antibodies. Non-mucoid strain (A) and mucoid (B) *S*. *aureus* strains (strains 8.1 and 8.2) were grown overnight in TSB. After washing, adherent bacteria were stained using SYTO 9 (green). PIA/PNAG was detected using ALEXA-568 labeled wheat germ agglutinin (red), which is a lectin binding to the N-acetylglucosaminyl backbone of PIA/PNAG. Zoom-in shows PIA/PNAG-embedded bacteria in the mucoid strain (B), while hardly any PIA/PNAG is detected with the non-mucoid isolate. White bar = 10 μm.

To further characterize biofilm formation of mucoid isolates in more detail, confocal laser scanning microscopy was performed. Staining of adherent bacteria using the LIVE/DEAD *Bac*Light kit detected no stable biofilm for non-mucoid isolates. Here, only occasionally cell clusters with different density and thickness were observed (Fig 3A). Intriguingly, bacteria were only loosely attached to each other, resulting in wobbling of bacterial consortia on mechanical stress, and the average biofilm thickness was low (<10 μM at 24 h). In contrast, the mucoid strain produced a stable biofilm, which revealed the typical mushroom-shaped multicellular structure (Fig 3B). Cells in this biofilm were tightly attached and a biofilm progression over time was present. While the surface was not entirely covered after 12 h, full coverage was reached after 24 h combined with a mushroom-shaped multicellular structure. The average height of the biofilm was 23 μM after 24 h. Furthermore, we observed an accumulation of dead cells inside the mushroom structures (Fig 3B). Staining of the cultures with antibodies against PIA revealed densely packed bacteria embedded in extracellular PIA/PNAG (Fig 4B) for the mucoid but not for the non-mucoid isolate (Fig 4A).

### Mucoid *S*. *aureus* isolates are protected against phagocytosis

**Fig 5:**
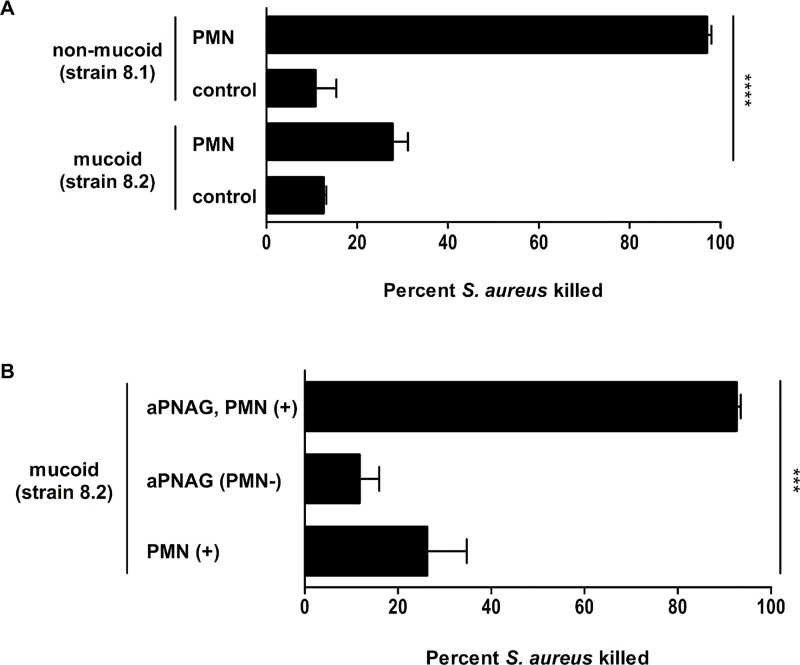
Opsonophagocytic killing of non-mucoid (strain 8.1) and mucoid *S*. *aureus* (strain 8.2) isolates by human neutrophils (PMNs) from healthy volunteers. The assay contained 2.5 x 10^6^ neutrophils, 5 x 10^5^ CFU *S*. *aureus*, 1% guinea pig serum (as a complement source), in a total volume of 500 μl MEM-BSA. Some assays included antibodies against PIA/PNAG raised in rabbits (1% PIA/PNAG). Control samples contained PMNs and *S*. *aureus* or antiserum and *S*. *aureus* alone with no PMNs. After 2 h of incubation, samples were vortexed and diluted in sterile deionized H_2_O to lyse the PMNs to release internalized bacteria. The lysate was plated on TSA plates to enumerate bacterial counts. Percent killing was calculated by the reduction in CFU/ml after 2 h compared with that at time zero. A) Significantly more non-mucoid compared to mucoid bacteria were killed in the presence of neutrophils (97% versus 28%, p<0.0001). In control wells, where neutrophils were absent, only 10% of bacteria died. B) By adding antibodies against PIA/PNAG to wells with mucoid isolates and neutrophils, 93% of bacteria were killed compared to only 26% killing of mucoid isolates in the presence of neutrophils but lacking the specific antibodies (p = 0.0002). All experiments were repeated three times. Results are shown as means and standard deviation. *** p-value ≤ 0.001; **** p-value ≤ 0.0001.

Since CF lung disease is characterized by enhanced inflammation with an accumulation of polymorphonuclear neutrophils (PMNs) [23], we assessed uptake and killing by fresh human PMNs of representative non-mucoid and mucoid isolates from patient 8 (strains 8.1 and 8.2, Table 2) in an *in vitro* assay. In contrast to the non-mucoid strain, which was readily killed by PMNs, the mucoid isolate resisted opsonophagocytic killing (Fig 5A). However, if antibodies against PIA/PNAG were added to the assay, robust phagocytic killing was achieved (Fig 5B). In contrast, CP8 antibodies, which interfere with the microcapsule of this isolate, which has been shown to belong to CP8, were poorly opsonic (S2 Fig) indicating that capsule formation had no impact on protection against neutrophilic killing. Therefore, these data revealed that opsonophagocytic killing of the mucoid *S*. *aureus* isolate was mediated by opsonic PIA/PNAG antibodies.

### Mucoid isolates carry a 5 bp deletion in the *icaR-icaA* intergenic region of the *ica* operon

Because mucoidy of the *S*. *aureus* isolates correlated with increased PIA/PNAG-mediated biofilm formation, we further characterized the *ica* operon of relevant *S*. *aureus* strains. As shown earlier by Jefferson et al. [20], a spontaneous laboratory mutant of *S*. *aureus* MN8 harbored a 5 bp deletion in the *icaR-icaA* intergenic region of the *ica* operon, resulting in constitutive expression of PIA/PNAG. Therefore, at first we sequenced the upstream *icaA* region of our mucoid isolates. All mucoid isolates carried exactly the same 5 bp deletion at the same position in this intergenic region. Interestingly, in three patients mucoid isolates co-colonized with non-mucoid *S*. *aureus* for periods up to 126 months (Table 1), which is exemplified more extensively for patient 8 in S1 Table.

### Non-mucoid strains with the 5 bp deletion carried compensatory mutations

**Fig 6:**
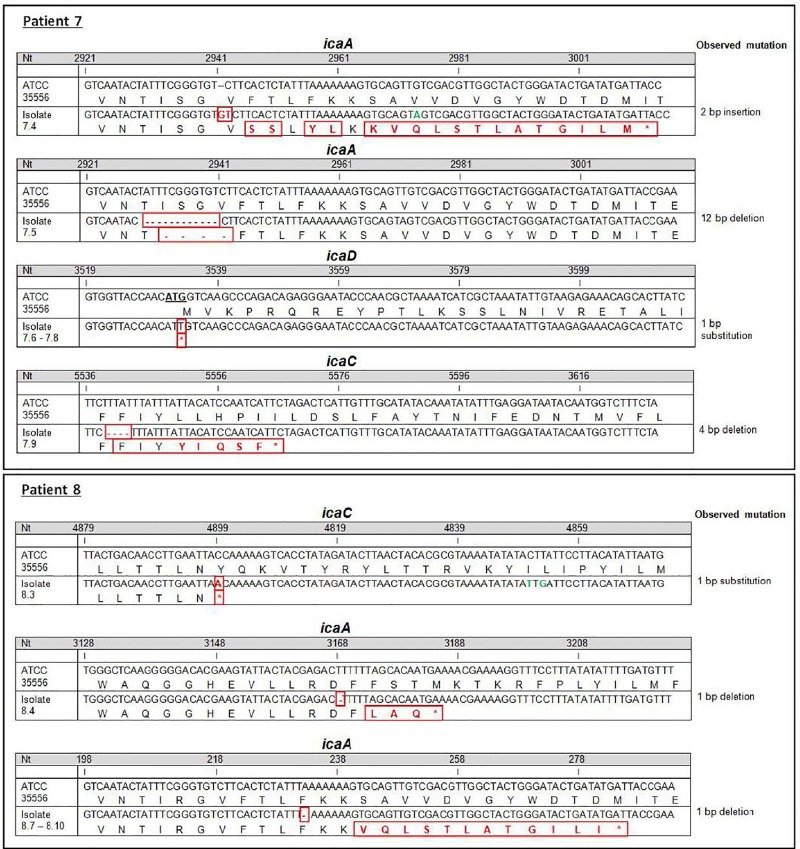
Mutations of non-mucoid *S*. *aureus* isolates with 5 bp deletions. During persistence, non-mucoid *S*. *aureus* isolates with 5 bp deletions and compensatory mutations in *icaA*, *D* and *C* were identified. The *ica*-sequences of 12 non-mucoid isolates with 5 bp deletions of patients 7 (A) and 8 (B) are illustrated. The numbering of bases and amino acids (aa) follows the sequences retrieved from *S*. *aureus* strain ATCC35556 (SA113). Changes of bases, amino acids and deletions are indicated in red color. Synonymous changes are indicated in green. *S*. *aureus* strains are identified by the number of the patient's visit and the date of isolation. For patient 7, the same mutation was identified for isolates from visits 14, 15 and 16 and for patient 8 for isolates from visits 27, 43, 49 and 51, respectively. Only the mutation for the first identified isolate is shown.

Surprisingly, from patients 7 and 8, who were persistently infected by mucoid *S*. *aureus* isolates for extended periods, non-mucoid isolates (n = 12), which carried the 5 bp deletion, were isolated. The non-mucoid isolates (Table 2) did not produce rough colonies on CRA or biofilm and therefore did not seem to produce high amounts of PIA/PNAG related biofilm anymore. To determine whether compensatory mutations in these late non-mucoid isolates with 5 bp deletions had occurred, we sequenced the entire *ica* operon and identified various mutations in *icaA* (n = 7), *icaD* (n = 3) and *icaC* (n = 2, Table 2, Fig 6).

**Fig 7:**
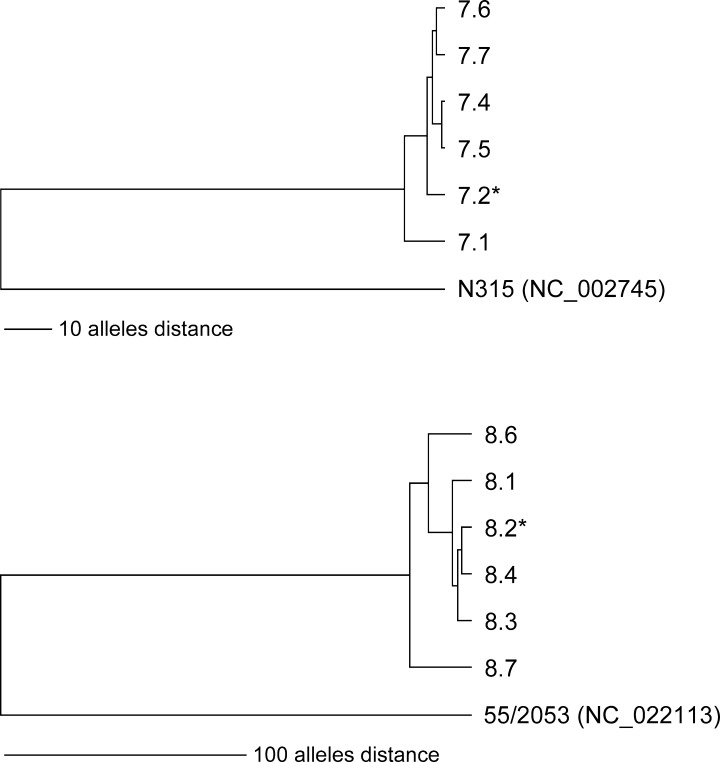
UPGMA-tree based on up to 1,861 cgMLST allelic profiles of *S*. *aureus* isolates of patients 7 and 8 (Table 2). The tree on the top shows the relationship of the six isolates of patient 7 including the reference strain N315, the tree on the bottom shows the relationship of the six isolates of patient 8 including the reference strain 55/2053. The scale, i. e. distances were given in absolute number of differing alleles. The strains marked with an asterisk exhibit a mucoid phenotype. For more information about isolates see Table 2.

Patient 7 carried *S*. *aureus* isolates with two different mutations in *icaA* that were detected in two different isolates recovered from one visit, the same mutation in *icaD* in three independent isolates during a period of 3 months, and mutations in *icaC* at one visit (Table 2). Patient 8 carried *S*. *aureus* with mutations in *icaC* identified at one visit and in *icaA* at five visits. Two of these isolates recovered in April 2007 (strain 8.4) and May 2008 (strain 8.7) carried different mutations in *icaA*. Six years later, three isolates with the same mutation in *icaA* as the isolate recovered in 2008 were cultured from March until November 2014 (strains 8.8, 8.9 and 8.10). Since there was a break of 6 years without the culture of isolates with such mutations, it is unlikely that these isolates persisted during this period without detection by culture. In line with this suggestion our whole genome sequencing data revealed that strain 8.7 is more distant from all other sequential isolates making it unlikely that this strain is the founding strain for strain 8.8 (Fig 7). Therefore, the same mutation in *icaA* emerged again in mucoid isolates. In summary, 10 of 12 isolates with 5 bp deletions but without PIA/PNAG hyper-expression showed mutations in *icaA* (n = 7) and *icaD* (n = 3).

### Whole genome sequencing (WGS) of non-mucoid and mucoid isolates of patients 7 and 8

To confirm that the mucoid isolates of patients 7 and 8 evolved from the non-mucoid *S*. *aureus* clone, we performed WGS of the first mucoid and nearest non-mucoid *S*. *aureus* isolates of these patients and also of the isolates with 5 bp deletion and compensatory mutations (Table 2). WGS and subsequent cluster analysis based on cgMLST (core genome MLST) allelic profiles of the each six strains of patient 7 and patient 8 exhibit a close relationship of all strains within each patient irrespective of the phenotype (Fig 7). Additional ANI (average nucleotide identity) calculation further corroborated this close relationship as all isolates of patient 7 had ANI values of 100% in pairwise comparisons. Similar ANI results were also observed for patient 8, where the ANI values were ≥ 99.99%.

### Complementation with vectors expressing wild-type *ica* genes confers the mucoid phenotype

**Fig 8:**
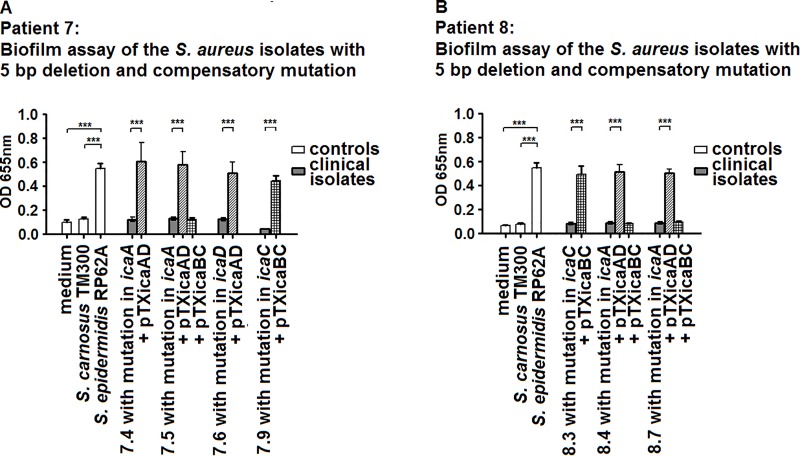
Biofilm formation of isolates with 5 bp deletion, compensatory mutations, which were complemented with the wild-type *ica* genes. Biofilm formation of the strains with compensatory mutations in *icaA*, *icaD* or *icaC* was abrogated. A) Isolates of patient 7. B) Isolates of patient 8. Transformation with a vector expressing the appropriate wild-type *ica* gene conferred biofilm formation.

To assess the role of the *icaA*, *icaD* and *icaC* mutations in non-mucoid *S*. *aureus* isolates with the 5 bp deletion, we used the xylose-inducible staphylococcal expression vectors pTX*icaBC* and pTX*icaAD*. Complementation of the isolates with the respective vectors restored the mucoid phenotype and allowed strong biofilm formation (Fig 8), corroborating that the compensatory mutations in *icaA*, *icaD* or *icaC* caused abrogation of biofilm and the non-mucoid phenotype.

### PIA/PNAG overexpression results in fitness costs in some but not all mucoid strains

**Fig 9:**
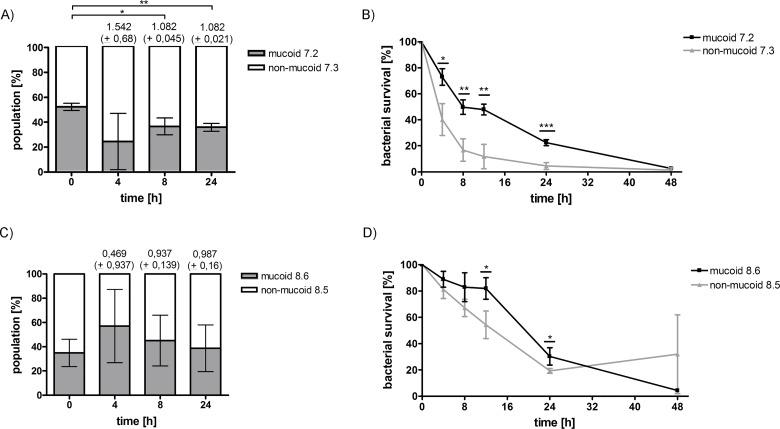
Competition and starvation experiments comparing non-mucoid and mucoid *S*. *aureus* isolates from patients 7 and 8. **A and C)** Competition growth experiments between the non-mucoid and mucoid strain pairs of patients 7 and 8 with fitness values indicated on top of the graph. During co-culture, the non-mucoid strain of patient 7 out-competed the mucoid strain, while for the strains of patient 8 no significant difference could be observed. **B and D)** The same strain pairs of patients 7 and 8 were compared concerning survival under nutrient limited conditions for 48h. For both patients, the mucoid strains survived significantly better during starvation compared to the non-mucoid strains. Data were generated by performing three biological with two technical replicates. Graphs show the average of three biological replicates with error bars indicating the standard deviation. Statistical analysis of the generated data was performed using an unpaired two-tailed t-test. * p-value ≤ 0.05; ** p-value ≤ 0.01; *** p-value ≤ 0.001.

Excessive production of PIA/PNAG might confer a fitness loss to mucoid *S*. *aureus* compared to non-mucoid isolates. To investigate this, we performed competition experiments between two non-mucoid and mucoid *S*. *aureus* strain pairs, which were isolated from patients 7 and 8. Whereas for patient 7, the non-mucoid strain outcompeted the mucoid isolate during co-culture (Fig 9A), for the strain pair of patient 8 no significant differences in fitness could be observed (Fig 9C). These results indicate that the emergence of a mucoid phenotype in vivo can, but is not necessarily associated with a fitness loss.

### Survival under limited nutrient conditions of *S*. *aureus*-isolates with mucoid and non-mucoid phenotype with and without the 5 bp deletion

To assess whether there is a difference of mucoid and non-mucoid isolates in terms of survival during starvation conditions that may occur at different sites in CF airways, we exposed the same non-mucoid/mucoid strain pairs as we used for the competition experiments to nutrient limited conditions lacking any carbon source [24]. Both mucoid strains of patients 7 and 8 were significantly better able to survive under these harsh conditions (Fig 9B and 9D). However, a non-mucoid strain without any mutations in the *ica* operon of patient 7, which was isolated at a later time point from the airways, showed a significantly better survival compared to the mucoid isolate (S3 Fig). Such results indicate that under starvation conditions mucoid isolates experience a survival advantage, which could be due to consumption of the extracellular polysaccharide. However, during in vivo persistence additional mutations might occur also in non-mucoid strains thereby facilitating survival under carbon-limited conditions.

**Fig 10:**
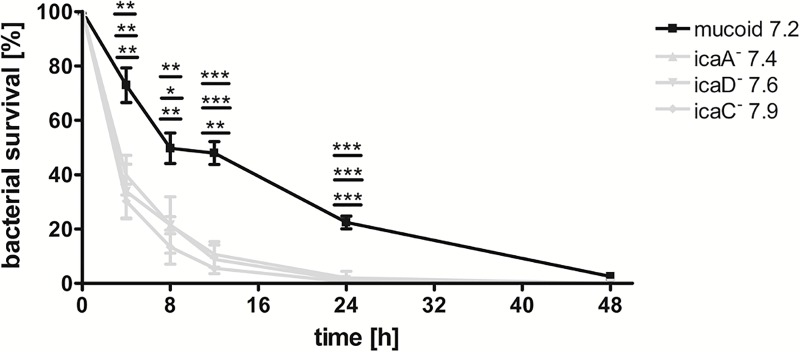
Growth of isolates with 5 bp deletion and compensatory mutations under nutrient limited conditions. Under nutrient limited conditions, all tested strains with 5 bp deletions and non-mucoid phenotype were less able to survive under these conditions compared to the isogenic mucoid isolate. Data were generated by performing two technical replicates per biological replicate. Graphs show the average of three biological replicates with error bars indicating the standard deviation. Statistical analysis was performed using an unpaired two-tailed t-test. * p-value ≤ 0.05; ** p-value ≤ 0.01; *** p-value ≤ 0.001.

Recently, a role for *icaC* as a target for phase variation has been suggested [24]. The authors showed that a strain with a 5 bp deletion and a compensatory mutation in *icaC* survived significantly better during starvation than the non-mucoid isolate with an intact *ica* operon. Therefore, we tested the survival of three non-mucoid strains with 5 bp deletions in comparison to the isogenic mucoid isolate during starvation. All strains with compensatory mutations were less able to survive under nutrient-limited conditions than the parental mucoid isolate (Fig 10) indicating that compensatory mutations in *icaA*, *icaD* or *icaC* are not advantageous for survival during starvation.

## Discussion

Although *S*. *aureus* chronically colonizes and infects the airways of CF patients, there are no previous reports of mucoid phenotypes of this pathogen recovered from the airways of these patients. Therefore, after occasional culture of mucoid *S*. *aureus* phenotypes from the specimens of some CF patients, we determined the prevalence of mucoid *S*. *aureus* isolates in *S*. *aureus*-positive patients from two independent prospective longitudinal studies, the persistence of mucoid isolates in the airways of these CF patients and the underlying molecular mechanism for mucoidy.

Our study revealed that mucoid *S*. *aureus* isolates i) can be isolated from app. 2% of CF patients with persistent *S*. *aureus* cultures without using a selective agar. Isolates with mucoid phenotypes might be missed during routine microbiological culture due to their distinct phenotype, which is sometimes only detectable by sub-culturing the mucoid isolate; ii) carried a 5 bp deletion in the *icaR-icaA* intergenic region, iii) evolve from non-mucoid *S*. *aureus* as indicated by molecular typing and WGS; iv) occurred during CF lung disease in many different genetic backgrounds of *S*. *aureus*; v) can persist in the airways for extended periods; vi) possess a survival advantage compared to the non-mucoid phenotype due to protection against neutrophil killing under aerobic conditions and may be optimized for survival under nutrient limited conditions and vii) that isolates with compensatory mutations in biofilm hyper-expressing clones in *icaA*, *icaD* or *icaC* emerge. Mucoid isolates were observed in 8 of 313 CF patients evaluated in two separate studies, and all investigated mucoid strains (n = 115) carried the same 5 bp deletion in the *icaR-icaA* intergenic region.

WGS of sequential *S*. *aureus* isolates of two CF patients confirmed that not only the mucoid isolates evolved from the non-mucoid strain, but also that the isolates with the non-mucoid phenotype and the 5 bp deletion, which additionally harbor compensatory mutations in *ica* genes, evolved from the mucoid isolates. The minor differences determined among the sequential isolates of the patients could be explained with microevolutionary events. It is known that approximately one mutation occurs in the core genome per six weeks [25]. Moreover, recent investigations demonstrated that even within *S*. *aureus* population of an individual with asymptomatic *S*. *aureus* nasal carriage a certain genomic diversity could be detected [26].Therefore, considering the selective pressure present in the hostile environment of the CF lung it is conceivable that sequential isolated *S*. *aureus* strains are not necessarily 100% identical as determined by WGS.

So far, only a single isolate with a 5bp deletion has been identified in *S*. *aureus* MN8m in vitro, but has never been reported to occur in vivo during *S*. *aureus* infections in patients [20]. Increased transcription of the *ica* operon was observed in strain MN8m. However, gel shift and DNase I footprint analyses revealed that this 5 bp motif within the *ica* promoter region did not affect binding of the repressor *icaR*. Therefore, the authors suggested that another protein must use this sequence to regulate *icaADBC* transcription.

Just recently, Yu L. et al. identified a new repressor of the *ica*-locus, which binds to exactly the intergenic region, which is absent in our mucoid isolates due to the 5 bp deletion thereby causing the hyper-expression of PIA/PNAG [27]. The authors identified this repressor in a clinical isolate, which exhibited also an unusual mucoid phenotype but did not reveal any changes in the *ica* operon, by microarray analysis and DNA sequencing. They named this repressor "rob", repressor of biofilm [27].

Compared to *S*. *epidermidis*, *S*. *aureus* produces only limited amounts of PIA/PNAG biofilm under aerobic conditions, whereas PIA/PNAG production is stimulated under anaerobic conditions, under the influence of SrrAB [18,19]. The *S*. *aureus* 5 bp deletion resulted in hyper-production of PIA/PNAG under aerobic conditions. Worlitzsch et al. reported that in the airways of CF patients anaerobic conditions are present in mucus plugs, resulting in biofilm formation in vivo [17]. However, within the environment of the airways there are different atmospheric levels of oxygen exposure. Therefore, hyper-production of PIA/PNAG under aerobic conditions could be advantageous for *S*. *aureus* in CF airways.

The CF lung disease is characterized by inflammation and recruitment of large numbers of neutrophils to the airways [23]. In an in vitro opsonophagocytic killing assay the mucoid isolate was protected against phagocytosis and killing by human neutrophils, whereas *S*. *aureus* with the non-mucoid phenotype was killed, confirming that biofilm overproduction is a protective mechanism against neutrophil killing. Ulrich et al. proposed a model for neutrophil killing of *S*. *aureus* under aerobic and anaerobic conditions in the airways [19]. Under aerobic conditions PIA/PNAG negative isolates are killed within neutrophils by reactive oxygen species (ROS) and defensins, while PIA/PNAG positive isolates are protected against killing by ROS but are killed by defensins [19]. However, our results suggest that hyper-production of PIA/PNAG not only protects against ROS, but also against killing by defensins. In light of the high rate of survival of mucoid *S*. *aureus* isolates during in vivo persistence in the lungs of some CF patients, hyper-production of biofilm may be an efficient strategy for *S*. *aureus* to avoid killing by PMNs under in vivo aerobic conditions.

Because biofilm formation requires additional energy output, it is possible that non-mucoid strains would be fitter than mucoid isolates. However, using two non-mucoid/mucoid strain pairs from two different patients our results did not necessarily support this hypothesis. In one strain pair the non-mucoid isolate out-competed the mucoid strain, while in the other strain pair the mucoid isolate was as much fit as the non-mucoid. Thus, mucoidy in clinical isolates might confer a fitness loss for some but not for all mucoid isolates. The comparable fitness of mucoid isolates compared to non-mucoid isolates might be due to additional mutations somewhere in the genome, which allow overcoming the energy costs due to excess biofilm formation. The results of the in vitro assays are also supported by our observation that mucoid isolates persisted for extended periods in vivo in the airways of CF patients.

Adaptation to limited nutrients also drives mutations in CF isolates as shown for *P*. *aeruginosa* [28]. Therefore, we also investigated the behavior of mucoid and non-mucoid isolates under carbon-limited conditions. Interestingly, two early mucoid isolates survived significantly better under these starvation conditions (Fig 9B and 9D) than the non-mucoid strains isolated at the same time (Table 2). Lack of carbon in the growth medium might be compensated in the mucoid isolates by consumption of the surrounding polysaccharide substrate, which then might lead to a better survival during starvation, while non-mucoid isolates die. However, we also show that in a later strain pair of patient 7 (S3 Fig) the non-mucoid isolate survived significantly better than the mucoid isolate. This result is in contrast to the results shown for the early mucoid/non-mucoid strain pairs and is most likely to further ongoing mutations in other parts of the genome in the non-mucoid isolate, which facilitate survival under starvation conditions without hyper-biofilm formation.

Interestingly, non-mucoid isolates that retained the 5 bp deletion were identified later during persistence in the airways. We hypothesized that a second mutation in the *ica* operon must have occurred in these strains, and therefore we sequenced the entire *ica* locus of these isolates. Sequencing results identified compensatory mutations, which were identified especially in *icaA* and *icaD* in 10 out of 12 isolates with the 5 bp deletion and a non-mucoid phenotype. The emergence of compensatory mutations in *ica* seems to be a reasonable strategy to avoid fitness costs required due to increased biofilm formation. However, most of the isolates with compensatory mutations were only isolated once or persisted only for a short period in the airways of CF patients, indicating that isolates with such compensatory mutations occur and might confer a short-term advantage but not an advantage for long-term persistence.

In line with this are the data of decreased survival of non-mucoid isolates with compensatory mutations in minimal medium. In our experiments we compared survival under nutrient limitation for three isolates with 5 bp deletions and compensatory mutations in *icaA* (2 bp insertion), *icaD* (1 bp substitution) and *icaC* (ttta-mutation) in comparison to the isogenic mucoid isolate. The observed decreased survival of these strains with compensatory mutations in *icaA*, *icaD* and *icaC* (Fig 10) are in contrast to the data by Brooks and Jefferson [24], who showed that their strain JB12, which possessed the same 5bp deletion and a ttta-mutation in *icaC* as our strain 7.9, survived better during nutrient starvation compared to the non-mucoid strain without 5bp deletion or the isogenic *ica*-deletion mutant. From their data Brooks and Jefferson suggested an important role for *icaA*, *icaD* and *icaB* genes in the absence of a functional *icaC* for bacterial survival under growth-limiting conditions [24]. Our contrasting results might be explained by different genetic backgrounds of the *S*. *aureus* strains tested or by the special conditions that *S*. *aureus* experiences during persistence in CF airways with ongoing mutations.

In summary, *S*. *aureus* isolates with mucoid colony morphology were observed during long-term persistence of *S*. *aureus* in the airways of several CF patients. Such isolates carried a 5 bp deletion in the *ica*R*-icaA* intergenic region of the *ica* operon and persisted in some patients for extended periods. Mucoid isolates were protected against phagocytosis and were partially optimized for survival under nutrient limited conditions. Later, non-mucoid *S*. *aureus* isolates with 5 bp deletion with compensatory mutations in various *ica* genes emerged without excess biofilm production. Such isolates were readily purged from the *S*. *aureus* population, while mucoid isolates still persisted. Thus, biofilm hyper-production of *S*. *aureus* represents an efficient strategy against phagocytic killing and also facilitates survival under starvation conditions thereby supporting long-term survival of *S*. *aureus* in CF airways, where neutrophils are highly predominant and nutrient starvation occurs.

In conclusion, the special conditions present in CF airways seem to facilitate ongoing mutations in the *ica* operon during persistence of *S*. *aureus*.

## Materials and Methods

### Study groups

Two different study groups were evaluated retrospectively for the occurrence of mucoid isolates. The first group consisted of CF patients with positive *S*. *aureus* cultures treated in the two CF centers in Münster, Germany (n = 118). We searched the microbiological database for patients with mucoid *S*. *aureus*. The second study group consisted of patients of a recently conducted prospective multicenter study (n = 195), in which only CF patients with positive *S*. *aureus* cultures the year before recruitment but without chronic *P*. *aeruginosa* infection were included (in revision).

### Strains and growth conditions

Several mucoid and non-mucoid *S*. *aureus* strains isolated from the respiratory tract of CF patients were characterized in this study (Tables 1, 2 and S1). The biofilm-negative *S*. *carnosus* TM300 and the biofilm-positive *S*. *epidermidis* RP62A (ATCC 35984) were used as controls. Staphylococci were grown in tryptic soy broth (TSB) (Becton, Dickinson and Company, Sparks, MD, USA), brain heart infusion (BHI) (Merck, Darmstadt, Germany), or on tryptic soy agar (TSA) (Becton, Dickinson and Company, Sparks, MD, USA) or Columbia blood agar plates and incubated for 24 h at 37°C. Congo red agar plates prepared with Columbia blood (CRA) were used to confirm the mucoid phenotype of *S*. *aureus* isolates [24]. When appropriate, erythromycin (10 μg/ml) was added to the medium. Induction was achieved by the addition of 0.5% xylose.

### Genotyping

*Cap-*typing was performed by multiplex PCR [29]; *spa*-typing and MLST were done as described before [30,31].

### Whole genome sequencing

To determine the clonal relationship of the strains of two patients with long-term persistence, we performed whole genome sequencing (WGS) of 12 *S*. *aureus* isolates of these patients as described [32] using the Illumina Nextera XT library preparation for a 250 bp paired-end sequencing run on a Miseq system (Illumina Inc., San Diego, CA, USA). Quality trimming, *de novo* assembly and subsequent core genome MLST (cgMLST) were performed as described recently [32]. For tree building using the Unweighted Pair Group Method with Arithmetic mean (UPGMA) method within the Ridom SeqSphere^+^ software (Ridom GmbH, Münster, Germany), the allelic profiles of the up to 1,861 cgMLST targets [33] were used applying the parameter “pairwise ignoring missing values”.

Moreover, we determined the average nucleotide identity (ANI) based on the *de novo* assembled contigs. Here, we used the ANI calculator (http://enve-omics.ce.gatech.edu/ani/ [34], which estimates the ANI using reciprocal best hits (two-way ANI) between two genomic datasets using default parameters. We assume that closely related isolates should exhibit > 99.9% ANI.

For comparison, genome sequences of *S*. *aureus* reference strains N315 (GenBank accession no. NC_002745; MLST ST5) and 55/2053 (NC_022113; MLST ST30) were used for patient 7 and patient 8, respectively, which reflect the most related *S*. *aureus* lineages to the patients’ isolates as determined by MLST.

All raw reads generated were submitted to the European Nucleotide Archive (http://www.ebi.ac.uk/ena/) under the study accession number PRJEB15647.

### Survival in minimal medium

To assess if there is a survival fitness of non-mucoid or mucoid isolates during starvation, bacteria were cultured in minimal medium according to Brooks et al. [24]. Briefly, bacteria were grown in 5 ml tryptic soy broth containing 1% glucose (TSBG) in 50 ml conical cell reactor tubes (Cellstar, No 227 245) at 160 rpm and 37°C. After 24h, cultures of mucoid isolates were supplemented with 5 μg/ml of Dispersin B (Kane Biotech) and cultivated for 30 min at 37°C to break up biofilm clusters. Bacteria were collected by centrifugation (4500 rpm, 7 min, RT) and pellets were resuspended in 5 ml MOPS minimal media (Teknova) lacking glucose to achieve a concentration of 10^8^ cells/ml. Bacteria were cultivated in 50 ml conical cell reactor tubes for up to 48h at 37°C and 160 rpm. After 0h, 4h, 8h, 12h, 24h and 48h cultures were vortexed, serially diluted and plated on Columbia blood agar incubated for 24h at 37°C to determine CFU/ml counts. Survival of isolates in minimal medium was calculated by comparing CFU/ml counts of respective time-points to the 0h CFU/ml counts (presented in %). Transformants harboring the xylose inducible plasmids pTXicaBC or pTXicaAD were cultivated as described above with the exception that the growth medium was supplemented with 10 μg/ml tetracycline and 0.5% (v/v) xylose. Statistical analysis was performed using an unpaired two-tailed t-test. * p-value ≤ 0,05; ** p-value ≤ 0,01; *** p-value ≤ 0,001.

### Competition experiments

Competition experiments were performed according to Brooks *et al*., 2014. Bacteria were grown in 5 ml TSBG in 12 ml tubes at 160 rpm and 37°C. Growth medium of mucoid isolates was supplemented with 5 μg/ml of Dispersin B (Kane Biotech) to prevent strong clustering due to excessive biofilm formation. After 24 h, bacteria were collected by centrifugation (4500 rpm, 7 min, RT) and pellets were resuspended in 5 ml TSBG. Cultures were diluted to a concentration of 10^8^ cells/ml and mucoid and non-mucoid isolates were mixed 1:1 in 50 ml conical cell reactor tubes (Cellstar). The medium was supplemented with 5 μg/ml of Dispersin B (Kane Biotech) to prevent biofilm formation and cultures were incubated for up to 24h at 37°C and 160 rpm. After 0h, 4h, 8h and 24h culture aliquots were vortexed, serially diluted and plated on CRA plates for CFU counting. CRA plates were incubated for 2–3 days at 37°C. Changes in population composition were calculated by comparing CFU/ml counts of mucoid or non-mucoid isolates to the CFU/ml counts of the whole population at the respective time-point (presented in %). Fitness of non-mucoid and mucoid isolates was calculated using the following function developed by Sander *et al*. M_t_ = ln [(n_t_/m_t_)/(n_t-1_/m_t-1_)^1/gen^] where n_t_ and m_t_ are the amount of non-mucoid and mucoid cells at a given time-point t, while n_t-1_ and m_t-1_ represent the amount of non-mucoid and mucoid cells at the preceding time-point [35]. For all isolates t_0_ was chosen to be the preceding time-point and fitness was calculated for the given time-points 4h, 8h and 24h according to http://textbookofbacteriology.net/growth_3.html. The function fit_t_ = 1+M_t_ was used to calculate the relative bacterial fitness. The fitness value is bigger than 1, if the non-mucoid isolate is fitter than the mucoid isolate. If there is no difference in fitness between the isolates, the value equals 1. If it is lower than 1, the non-mucoid isolate has a reduced fitness compared to the mucoid isolate. Statistical analysis of the generated data was performed using an unpaired two-tailed t-test. * p-value ≤ 0,05; ** p-value ≤ 0,01; *** p-value ≤ 0,001.

### Biofilm assay

A static biofilm assay followed by crystal violet staining was modified from a previous report [36]. Briefly, an overnight culture of *S*. *aureus* was diluted 200-fold with BHI containing 0.25% glucose, of which 200 μl were added to the wells of a 96-well polystyrene microtiter plate (Greiner Bio-One, Frickenhausen, Germany) and incubated for 24 h at 37°C. To determine the amount of biofilm produced by each clinical *S*. *aureus* isolate, all plates were washed three times with PBS and the on the bottom adhering biofilms were stained with 1% crystal violet for 15 min. Following three further washing steps with PBS, biofilms were solubilized in 100 μl ethanol-acetone (80:20). The absorbance was determined at OD_655nm_ with a microtiter plate reader (Bio-Rad, Hercules, CA, USA). In parallel experiments, the nature of the formed biofilms was analyzed. Therefore, microtiter plates that were incubated for 24 h at 37°C were treated either with 100 μl sodium-metaperiodate in water (40 mM; AppliChem, Darmstadt, Germany), DNase I in 150 mM NaCl/1mM CaCl_2_(100 μg/ml; Roche, Mannheim, Germany) or proteinase K in 10 mM Tris-HCl (pH 7.5) (100 μg/ml; MP Biomedicals, Santa Ana, California, USA) for 3 h at 37°C. After three washing steps with PBS, biofilms were stained with crystal violet, washed again with PBS and solubilized in ethanol-acetone as described above. To confirm accuracy and reproducibility, each isolate was investigated in three biological replicates, always in eight wells per microtiter plate. *S*. *epidermidis* RP62A, which is known to form a strong biofilm with PIA as the major component [37] served as positive control, and the biofilm-negative and PIA-*icaADBC*-negative *S*. *carnosus* TM300 served as a negative control [38].

### Confocal laser scanning microscopy

*S*. *aureus* isolates were grown overnight in 300 μl TSB under static conditions in six-well cell culture plates (μ-Dish, Ibidi, Munich, Germany). In some experiments Alexa-568 labelled Wheat germ agglutinin or antibodies against PIA were added to the growth medium. Non-adherent cells were removed by washing with PBS and bacteria were stained using live staining (Live/dead staining, Molecular Probes). Confocal image acquisition was performed on a Zeiss Axiovert 200M inverted microscope equipped with a Yokogawa CSU-22 confocal head and a Hamamatsu C9100-02 EM-CCD camera. Images were taken with a Zeiss Plan Apochromat 63x/1.4 Ph3 Oil objective. Improvision Velocity software was used for image acquisition and quantification.

### Quantification of bacterial polysaccharide production

Capsule was semi-quantified by colony immunoblots as described before [22,39], and bacterial polysaccharides (CP and PIA/PNAG) were quantified by enzyme-linked immunosorbent (ELISA) inhibition assays [40,41]. Briefly, 96-well plates were coated overnight at 4°C with purified PNAG (1 μg/ml) or with CP5 or CP8 (4 μg/ml) coupled to poly-*L*-lysine by the cyanuric acid chloride method. The microtiter plate was washed and blocked with 0.05% skim milk. *S*. *aureus* strains were harvested, washed in phosphate buffer, and then trypsinized (1 mg trypsin/ml of 0.1 M phosphate buffer, pH 8) for 60 min at 37°C to remove protein A. After washing, the bacterial suspensions were serially diluted, and the bacterial concentrations were verified by plating on tryptic soy agar plates. Polyclonal polysaccharide-specific antiserum was diluted and incubated overnight at 4°C with serial dilutions of the bacteria or purified polysaccharide (standard curve ranging from 1 μg/ml to 1 ng/ml). Samples were centrifuged, and the absorbed serum samples (supernatants) were added to the coated microtiter plates. Following a 2-h incubation with absorbed or unabsorbed serum samples, the plates were washed with PBS/Tween, and alkaline phosphatase-conjugated protein A/G (Thermo Scientific; 1:3000) was added to each well. After a 2-h incubation at ambient temperature, the plate was washed, and the substrate *p*-nitrophenyl phosphate was added. When the wells containing unabsorbed serum samples reached an OD_405 nm_ of ~2.0, the plate was read on a Bio-TEK Power Wave HT ELISA reader. The concentration of each sample (CFU/ml) that resulted in 50% inhibition of antibody binding (IC_50_) was determined, and the polysaccharide content of the sample was calculated from the standard curve.

### Opsonophagocytic killing assays

The opsonophagocytic killing (OPK) activity of human polymorphonuclear neutrophils (PMNs) was performed and analyzed as described [42].

### Transformation with expression vectors

The staphylococcal expression vectors pTXi*caBC* and pTX*icaAD* [43] were used to complement the *S*. *aureus* strains with 5 bp deletions and non-mucoid phenotype. The plasmids were transformed into the cells by electroporation using standard procedures as described [44]. Plasmid purification was performed following the PrepEase Quick MiniSpin Plasmid kit protocol (Affymetrix).

### DNA methods, molecular techniques

Genomic DNA (gDNA) was isolated according to the manufacturer instructions of the QiAamp DNA Mini Kit (Qiagen, Hilden, Germany). For sequencing of the *icaADBC* operon, amplified PCR products were purified with the QIAquick PCR Purification Kit (Qiagen, Hilden, Germany) according to the instructions of the manufacturer. Sequence analysis was conducted using the software Clone Manager Suite 7 (Scientific & Educational Software, Durham, NC) and the reference sequence of *S*. *aureus* ATCC35556. Primers are shown in Table 3.

**Table 3:** List of primer pairs used for the amplification of the four genes of the *ica*-operon (*icaA*, *icaD*, *icaB and icaC*), the upstream localized gene *icaR* (coding for the regulator), and the intermediate promoter region.

Primer name	5´->3´ - Sequence	Amplification length (bp)
Ica_Promoter-for	TACCGTCATACCCCTTCTCT	369
Ica_Promoter-rev	TGCCTTCTAATTCATCCACA	
Ica_Repressor-for	CAATATCGATTTGTATTGTCAACTTT	798
Ica_Repressor-rev	GGTTGTAAGCCATATGGTAATTGA	
IcaA1-for	CCCCCTACTGAAAATTAATCACA	808
IcaA1-rev	GTAGCCAACGTCGACAACTG	
IcaA2-for	TGCTGGCGCAGTCAATACTA	857
IcaA2-rev	GCGAAAATGCCCATAGTTTC	
IcaD/B1-for	CGCAGCAGTAGTTCTTGTCG	802
IcaD/B1-rev	ATAAACCCAGTCGCCGGTAT	
IcaB2/C1-for	GATCATATTGCCTGTAAG	657
IcaB2C1-rev	ATCAAGCCATAAGGATAG	
IcaC2-for	CTGGGTTATGGGAATTTG	879
IcaC2-rev	ATAGTGTAGCACGGTATC	
IcaC3-for	CCTATTAGGTCAATGGTATGG	838
IcaC3-rev	CCATTGGCATTTACGAAG	
Ica x1-for	CGCCTATGTCATGATTTACC	463
Ica x1-rev	AATTTGGAGCAGTGGAAG	
Ica x2-for	ATGCTTTCAAATACCAACTTTC	458
Ica x2-rev	GATTATTGATAACGCAATAACC	
Ica x3-for	AAATTCCTCAGGCGTATTAG	544
Ica x3-rev	ACCGACAATCCAGTAAATAG	
Ica x4-for	CGAAAGGTAGGTAAAGAAATTG	300
Ica x4-rev	ATGAGTTCTGCTGTATTATCTG	
Ica x5-for	GACATAAATGTGGATGAATTAG	566
Ica x5-rev	ATATCTTCGGTAATCATATCAG	
Ica x6-for	ATTGCAGTTTCTTGGAAATTG	470
Ica x6-rev	ACTACTGCTGCGTTAATAATC	
Ica x7-for	GTGAGATGGGCTCAAGGG	430
Ica x7-rev	CTCTGTCTGGGCTTGACC	
Ica x8-for	AAGCCCAGACAGAGGGAATAC	369
Ica x8-rev	GACCATCCAGTGTGCTTACAG	
Ica x9-for	TACCGTGCTACACTATTATCCC	663
Ica x9-rev	TCCCATTGGCATTTACGAAG	
Ica x10-for	GAAAGCTTCTGAAGCTACAATC	809
Ica x10-rev	ATAATAGTGTAGCACGGTATCG	
Ica x11-for	TCACTCCGAACTCCAATG	603
Ica x11-rev	CGCTGTGTTGTTCGTAAAG	
Ica x12-for	TTTCGTCGATTTACAAG	651
Ica x12-rev	AAGAAGTTTGCTGTTATG	

The primer pairs were created using the reference sequence of the *S*. *aureus* strain ATCC35556 and the program Clone Manager.

## Supporting Information

S1 Fig **Quantification of PIA/PNAG (A) and capsule polysaccarides (B).** Bacterial polysaccharides (CP and PIA/PNAG) were quantified by enzyme-linked immunosorbent (ELISA) inhibition assays. 96-well plates were coated with purified PNAG (1 μg/ml) or with CP5 or CP8 (4 μg/ml). Bacterial suspensions were diluted. Polyclonal polysaccharide-specific antiserum was diluted and incubated overnight at 4°C with serial dilutions of the bacteria or purified polysaccharide (standard curve ranging from 1 μg/ml to 1 ng/ml). Samples were centrifuged, and the supernatants were added to the coated microtiter plates. The concentration of each sample (CFU/ml) that resulted in 50% inhibition of antibody binding (IC_50_) was determined, and the polysaccharide content of the sample was calculated from the standard curve. Information of used *S*. *aureus* isolates is given in S1 Table.
(TIF)

S2 Fig See Figure legend 5. (TIF)

S3 Fig Another non-mucoid/mucoid strain pair of patient 7 was compared concerning survival under nutrient limited conditions for 48h. In contrast to the results shown in Fig 9, in this strain pair, which was isolated at a later time point from the airways of this patient, the non-mucoid strains survived significantly better during starvation compared to the mucoid strain indicating that further mutations somewhere in the genome must be responsible for this phenotype. Data were generated by performing three biological with two technical replicates. Graphs show the average of three biological replicates with error bars indicating the standard deviation. Statistical analysis of the generated data was performed using an unpaired two-tailed t-test. * p-value ≤ 0.05.
(TIF)

S1 Table Phenotypic and genotypic characteristics of sequential *S*. *aureus* isolates of patient 8. ^1^Some strains with numbers were introduced in Table 2; strains with M and numbers are sequential isolates of patient 8.
^2^Production of capsules was semi-quantified on colony immunoblots by the intensity of the CP8 antibody reaction ranging from 0 to 4.
^3^Presence or absence of the 5-bp deletion in the promoter region of the *ica* operon was determined by sequencing.
^4^Clonality of isolates determined by *spa* sequencing
^5^+/- very low reaction
(DOCX)
