# Supplementary figures and images for: Dynamic in vivo mutations within the ica operon during persistence of Staphylococcus aureus in the airways of cystic fibrosis patients
Source: PLoS Pathog. 2016 Nov 30;12(11):e1006024. doi: 10.1371/journal.ppat.1006024 (PMC5130281; doi:10.1371/journal.ppat.1006024)

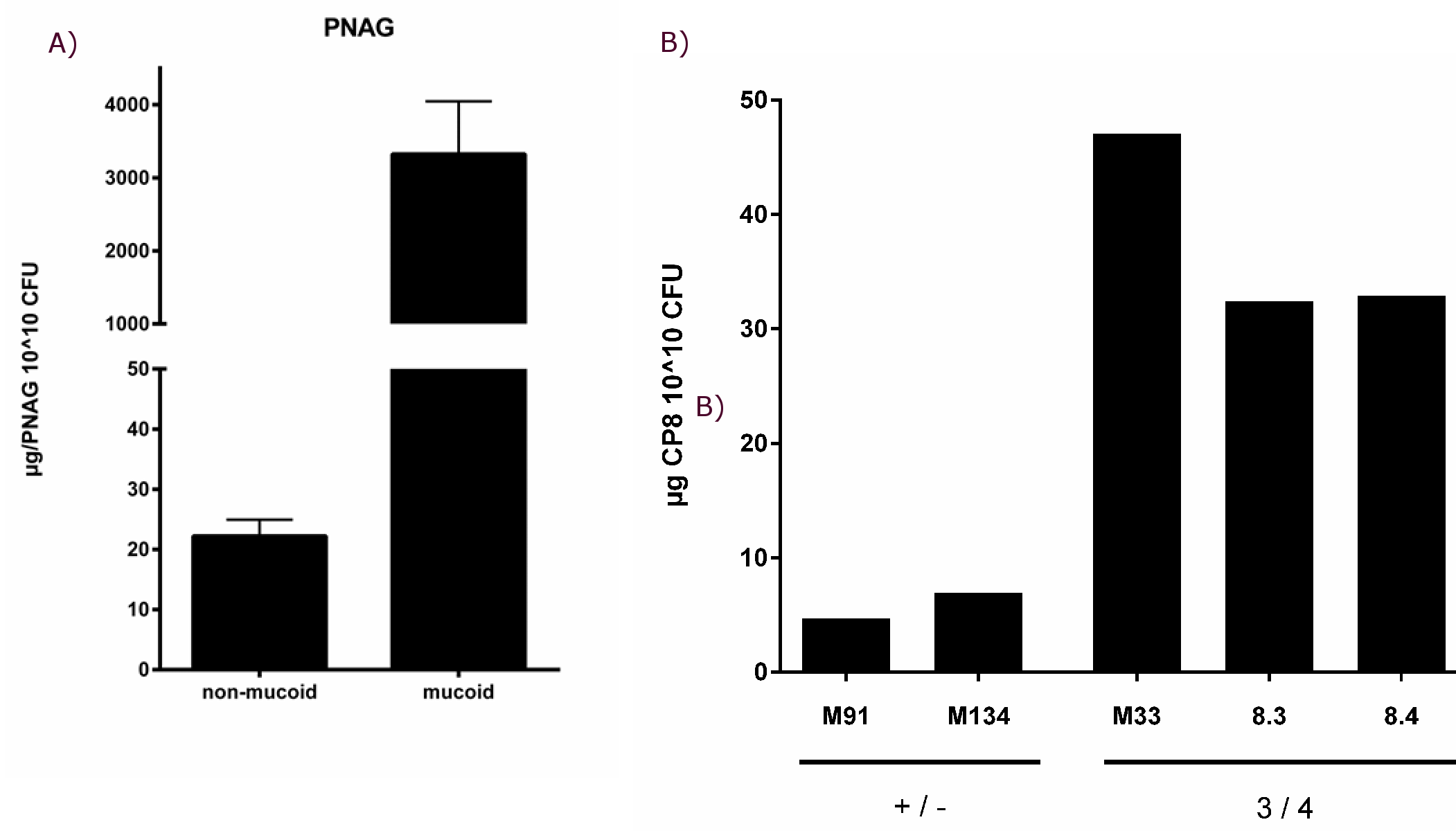

Supplement: S1 Fig — Quantification of PIA/PNAG (A) and capsule polysaccarides (B). Bacterial polysaccharides (CP and PIA/PNAG) were quantified by enzyme-linked immunosorbent (ELISA) inhibition assays. 96-well plates were coated with purified PNAG (1 μg/ml) or with CP5 or CP8 (4 μg/ml). Bacterial suspensions were diluted. Polyclonal polysaccharide-specific antiserum was diluted and incubated overnight at 4°C with serial dilutions of the bacteria or purified polysaccharide (standard curve ranging from 1 μg/ml to 1 ng/ml). Samples were centrifuged, and the supernatants were added to the coated microtiter plates. The concentration of each sample (CFU/ml) that resulted in 50% inhibition of antibody binding (IC50) was determined, and the polysaccharide content of the sample was calculated from the standard curve. Information of used S. aureus isolates is given in S1 Table. (TIF) [file ppat.1006024.s001.tif]

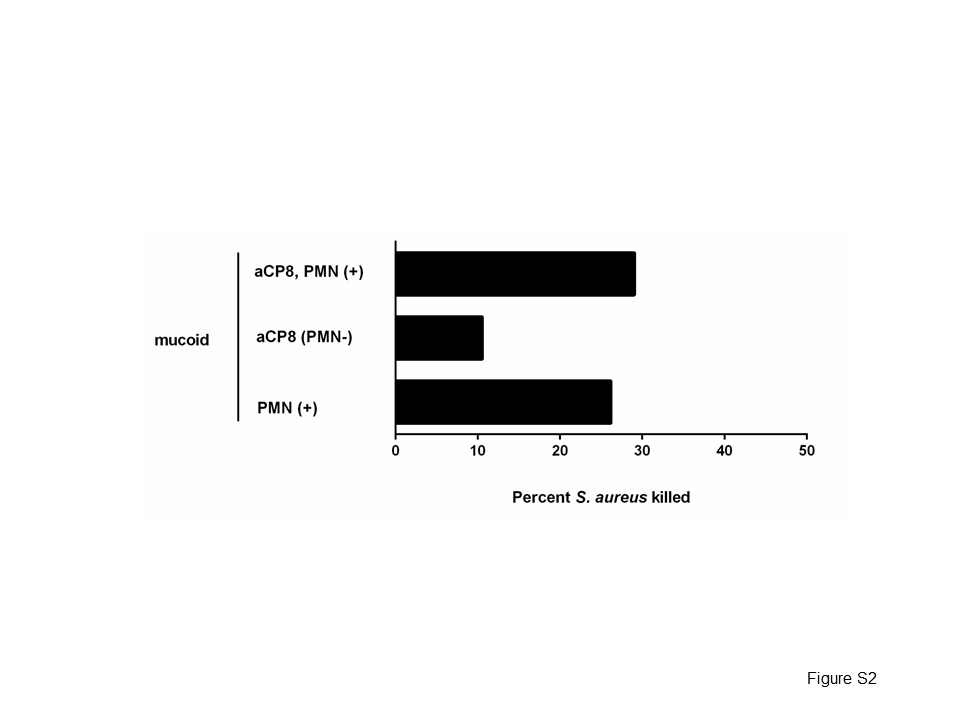

Supplement: S2 Fig — (TIF) [file ppat.1006024.s002.tif]

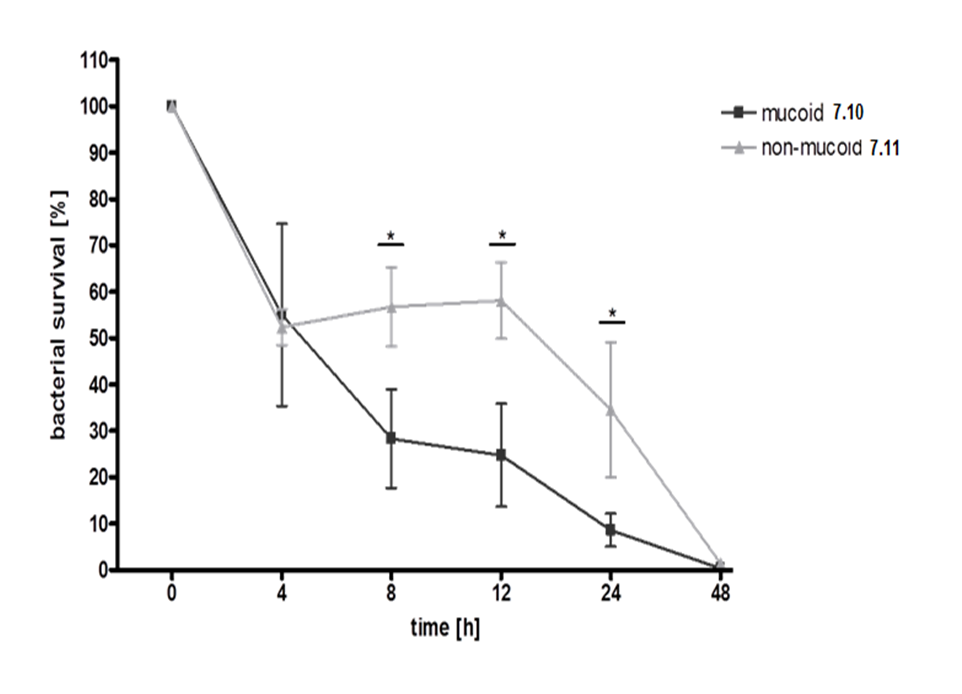

Supplement: S3 Fig — In contrast to the results shown in Fig 9, in this strain pair, which was isolated at a later time point from the airways of this patient, the non-mucoid strains survived significantly better during starvation compared to the mucoid strain indicating that further mutations somewhere in the genome must be responsible for this phenotype. Data were generated by performing three biological with two technical replicates. Graphs show the average of three biological replicates with error bars indicating the standard deviation. Statistical analysis of the generated data was performed using an unpaired two-tailed t-test. * p-value ≤ 0.05. (TIF) [file ppat.1006024.s003.tif]
