# Supplementary material for: Dynamic in vivo mutations within the ica operon during persistence of Staphylococcus aureus in the airways of cystic fibrosis patients
Source: PLoS Pathog. 2016 Nov 30;12(11):e1006024. doi: 10.1371/journal.ppat.1006024 (PMC5130281; doi:10.1371/journal.ppat.1006024)
Supplement: S1 Table — 1Some strains with numbers were introduced in Table 2; strains with M and numbers are sequential isolates of patient 8. 2Production of capsules was semi-quantified on colony immunoblots by the intensity of the CP8 antibody reaction ranging from 0 to 4. 3Presence or absence of the 5-bp deletion in the promoter region of the ica operon was determined by sequencing. 4Clonality of isolates determined by spa sequencing 5+/- very low reaction (DOCX) [file ppat.1006024.s004.docx]

**Table S1. Phenotypic and genotypic characteristics of sequential *S. aureus* isolates of patient 8.**

| **Strain design-ation** |  | **date of** | **phenotypes** | | **CP8^2^** | **5-bp** | ***spa*** |
| --- | --- | --- | --- | --- | --- | --- | --- |
|  | **specimen** | **isolation** | **mucoid** | **normal** |  | **Deletion^3^** | **Type^4^** |
| 8.1 | sputum | 2005 |  | + | 1 |  | t618 |
| M8 | sputum | 2005 | + |  | 4 | + | t618 |
| 8.2 | sputum | 2005 | + |  | 2 | + | t618 |
| M11 | nasal | 2005 |  | + | 2.5 |  | t618 |
| M12 | nasal | 2005 |  | + | 3 |  | t618 |
| M32 | nasal | 2006 |  | + | 2 |  | t618 |
| M33 | sputum | 2006 | + |  | 4 | + | t618 |
| M38 | sputum | 2006 | + |  | 4 | + | t618 |
| 8.3 | nasal | 2006 |  | + | 3 | + | t618 |
| M73 | sputum | 2007 | + |  | 4 | + | t618 |
| 8.4 | sputum | 2007 |  | + | 3 | + | t618 |
| M88 | throat | 2008 | + |  | 4 | + | t618 |
| M90 | throat | 2008 | + |  | +/-^5^ | + | t618 |
| M91 | throat | 2008 | + |  | +/- | + | t618 |
| M93 | sputum | 2008 |  | + | 3 | + | t618 |
| M94 | sputum | 2008 | + |  | +/- | + | t618 |
| M95 | sputum | 2008 | + |  | +/- | + | t618 |
| M127 | sputum | 2009 | + |  | 4 | + | t618 |
| M134 | sputum | 2010 | + |  | +/- | + | t618 |
| M138 | sputum | 2010 | + |  | + | + | t618 |

^1^Some strains with numbers were introduced in Table 2; strains with M and numbers are sequential isolates of patient 8.

^2^Production of capsules was semi-quantified on colony immunoblots by the intensity of the CP8 antibody reaction ranging from 0 to 4.

^3^Presence or absence of the 5-bp deletion in the promoter region of the *ica* operon was determined by sequencing.

^4^Clonality of isolates determined by *spa* sequencing

^5^+/- very low reaction
